# AZD1208, a Pan-Pim Kinase Inhibitor, Has Anti-Growth Effect on 93T449 Human Liposarcoma Cells via Control of the Expression and Phosphorylation of Pim-3, mTOR, 4EBP-1, S6, STAT-3 and AMPK

**DOI:** 10.3390/ijms20020363

**Published:** 2019-01-16

**Authors:** Anil Kumar Yadav, Vinoth Kumar, David Bishop Bailey, Byeong-Churl Jang

**Affiliations:** 1 Department of Molecular Medicine, College of Medicine, Keimyung University, 1095 Dalgubeoldaero, Dalseo-gu, Daegu 42601, Korea; aydaegu@gmail.com (A.K.Y.); vinbavin@gmail.com (V.K.); 2 Comparative Biomedical Sciences, Royal Veterinary College, London NW1 0TU, UK; dbishopbailey@rvc.ac.uk

**Keywords:** AZD1208, Pim-3, AMPK, STAT-3, 93T449

## Abstract

Overexpression of Pim kinases has an oncogenic/pro-survival role in many hematological and solid cancers. AZD1208 is a pan-Pim kinase inhibitor that has anti-cancer and anti-adipogenic actions. Here, we investigated the effects of AZD1208 on the growth of 93T449 cells, a differentiated human liposarcoma cell line. At 20 µM, AZD1208 was cytotoxic (cytostatic) but not apoptotic, reducing cell survival without DNA fragmentation, caspase activation or increasing cells in the sub G1 phase; known apoptotic parameters. Notably, AZD1208 reduced phosphorylation of signal transducer and activator of transcription-3 (STAT-3) in 93T449 cells. STAT-3 inhibition by AG490, a JAK2/STAT-3 inhibitor similarly reduced cell survival. AZD1208 down-regulated phosphorylation of mammalian target of rapamycin (mTOR) and ribosomal S6 while up-regulated eukaryotic initiation factor-2α (eIF-2α). In addition, AZD1208 induced a LKB-1-independent AMPK activation, which was crucial for its cytostatic effect, as knock-down of AMPK greatly blocked AZD1208s ability to reduce cell survival. AZD1208 had no effect on expression of two members of Pim kinase family (Pim-1 and Pim-3) but inhibited phosphorylation of 4EBP-1, a downstream effector of Pim kinases. Importantly, a central role for Pim-3 in the actions of AZD1208 was confirmed by knock-down, which not only reduced 93T449 cell survival but also led to the inhibition of 4EBP-1, mTOR, eIF-2α and STAT-3, along with the activation of AMPK. In summary, this is the first report demonstrating that AZD1208 inhibits growth of liposarcoma cells and that this activity is mediated through Pim-3 kinase, STAT-3, mTOR, S6 and AMPK expression and phosphorylation pathways.

## 1. Introduction

Soft tissue sarcomas are a heterogeneous group of solid malignant tumors having various histologies and commonly characterized by aggressive characteristics locally and in distant metastases [1,2]. Notably, 13,040 new cases of soft tissue sarcomas have been reported so far in the United States in 2018 [3]. Liposarcoma represents one of the most common subtypes of soft tissue sarcoma and are associated with considerable morbidity and mortality and a particularly poor prognosis depending on histological subtype, anatomical location and tumor burden [4,5,6]. Despite the development and clinical utilization of new-targeted chemotherapeutic agents, improved radiation targeting and tissue sparing approaches and new surgical techniques, unfortunately, only a minimal increase in overall survival of sarcoma patients has been demonstrated in the last two decades [7]. Thus, there still remains a great need for more effective and non-toxic therapeutics to enhance locoregional disease control as well as overall survival in sarcoma patients.

The provirus integration site for moloney murine leukemia virus (Pim) kinases, composing of Pim-1, Pim-2 and Pim-3, are active serine (S)/threonine (T) kinases which are highly homologous with overlapping functions and substrate specificities [8]. They modulate the activity of a variety of downstream effector proteins, such as, eukaryotic translation initiation factor 4E binding protein-1 (4EBP-1), mammalian target of rapamycin (mTOR), the 40S ribosomal subunit S6 protein, p70 S6 kinase (p70S6K), signal transducers and activators of transcription-3 (STAT-3), AMP-actuated protein kinase (AMPK), involved in the control of cell cycle, survival, transcription, translation, drug resistance and signaling within the microenvironment [8,9,10,11]. Unlike other kinases, Pim kinases are constitutively expressed and activated and tightly controlled. However, there is accumulating evidence that Pim kinases are overexpressed in many hematological malignancies [12,13,14] and solid tumors [15,16] and their overexpression plays a oncogenic/pro-survival role in such tumors. Due to this oncogenic/pro-survival role in cancer biology, Pim kinases are an emerging target for anti-cancer therapy. The attribution of oncogenic activity to all three Pim isoforms and the potential for redundancy argues for the development of inhibitors capable of targeting all family members. Gene knockout studies also have demonstrated that mice deficient for all three Pim kinases are viable and fertile [17], which supports the tolerability of pan-Pim kinase inhibition. Pim kinases contain a unique ATP-binding pocket, which has resulted in the development of highly selective pan-Pim inhibitors such as AZD1208 [18,19].

AZD1208 is a highly selective and orally available inhibitor for all three members of Pim kinase family and is known for its anti-cancerous activity in a broad range of cancer cell lines [20,21,22,23,24]. Recently, we have demonstrated that AZD1208 inhibits adipogenesis of 3T3-L1 adipocytes, addressing its anti-obesity effect [25]. Interestingly, there is further evidence demonstrating Pim kinases are important mediators of adipocyte differentiation [26] and considered as a marker of adipocytic differentiation in adipocytic neoplasm [27]. At present, little is known about the expression and role of Pim kinases in adipocytic tumors. In this study, we investigated the effects of AZD1208 on the growth of 93T449 cells, a fully differentiated human adipocytic tumor cell line. Here we report firstly that AZD1208 inhibits growth of 93T449 cells, which is mediated through Pim kinases regulation of 4EBP-1, STAT-3, mTOR, S6, eIF-2α and AMPK.

## 2. Results

### 2.1. AZD1208 Strongly Inhibits Growth of 93T449 Human Liposarcoma Cells

**Figure 1:**
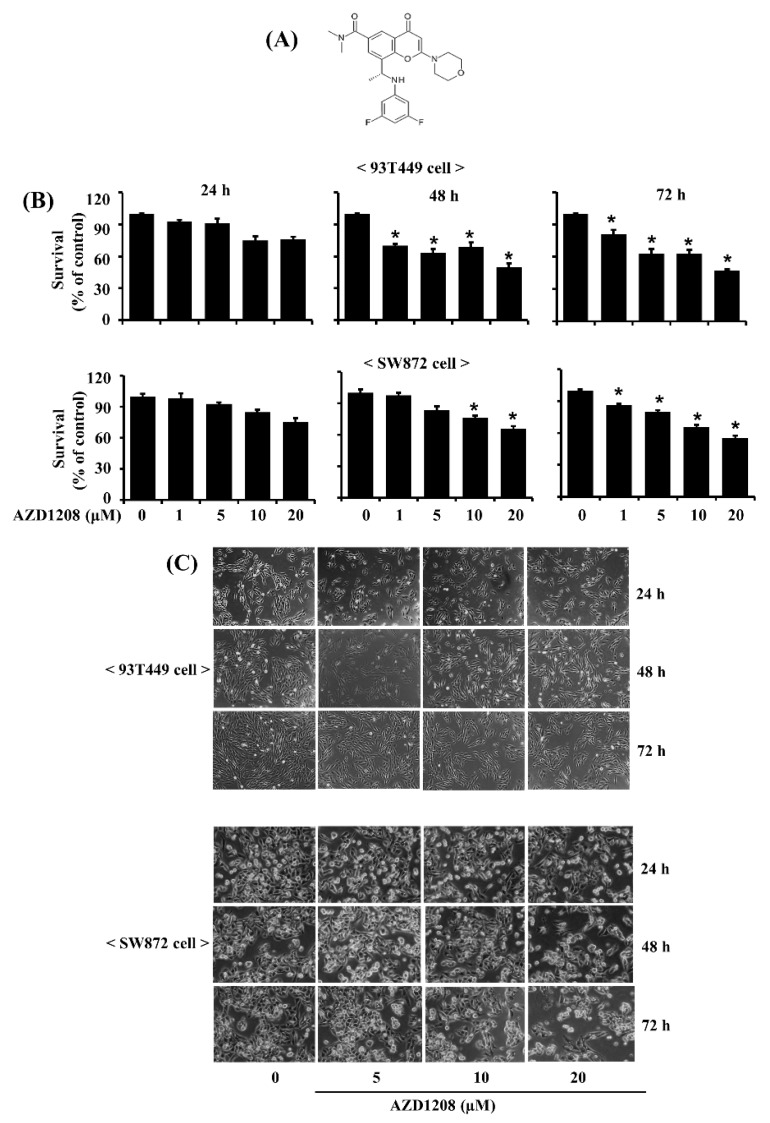
Effect of AZD1208 on survival of 93T449 and SW872 cells. (**A**) The chemical structure of AZD1208. (**B**) 93T449 and SW872 cells were treated with AZD1208 or vehicle control (DMSO; 0.1%) at the indicated concentrations and for the indicated times. The numbers of surviving cells were measured by cell count assay. The cell count assay was performed in triplicate. Data are means ± SE of three independent experiments. * *p* < 0.05 compared to the value of AZD1208 free control at the indicated time. (**C**) 93T449 and SW872 cells were treated with AZD1208 or vehicle control (DMSO) for the indicated times. Images of the conditioned cells were obtained by phase contrast microscopy, 200 ×. Each image is a representative of three independent experiments.

Initially, we investigated the effects of AZD1208 (Figure 1A) at different concentrations (1, 5, 10 or 20 µM) and times (24, 48 or 72 h) on the growth of 93T449 and SW872 cells by cell number (Figure 1B) and morphology (Figure 1C). AZD1208 reduced survival of 93T449 and SW872 cells in a concentration and time-dependent manner (Figure 1). Microscopic observation further revealed that AZD1208 caused a concentration and time-dependent reduction of 93T449. Next, to see whether the AZD1208’s growth inhibition is limited to 93T449 cells, we tested the effects of AZD1208 on the growth of SW872 cells, another human liposarcoma cell line. AZD1208 also caused a concentration- and time-dependent decrease in the survival of SW872 cells. These results verify that the observed response to AZD1208 is not cell line dependent. Because of stronger growth inhibitory effect on 93T449 at 20 µM, we decided to focus on this concentration of AZD1208 and 93T449 for further studies.

### 2.2. AZD1208 Does Not Induce Apoptosis of 93T449 Human Liposarcoma Cells

**Figure 2:**
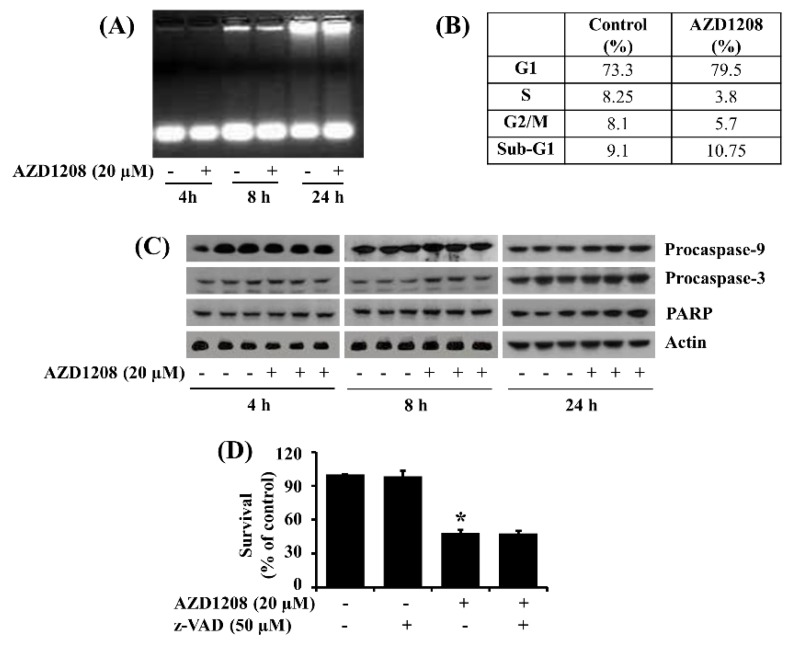
Effect of AZD1208 on apoptosis of 93T449 cells. (**A**) 93T449 cells were treated with AZD1208 (20 µM) or vehicle control (DMSO) for the times indicated. At each time point, extra-nuclear fragmented DNA from the conditioned cells was extracted and analyzed on a 1.7% agarose gel. The image is a representative of three independent experiments. (**B**) 93T449 cells were treated with AZD1208 (20 µM) or vehicle control (DMSO) for 24 h. The conditioned cells were harvested and subjected to fluorescence-activated cell sorting (FACS) analysis for measuring the population of sub G1 phase. The tables represent the fraction of apoptotic cells. (**C**) 93T449 cells were treated with AZD1208 (20 µM) or vehicle control (DMSO) in triplicate experiments for the times designated. At each time point, whole cell lysates were prepared and analyzed for procaspase-9, procaspase-3, PARP or β-actin expression or cleavage by Western blotting. (**D**) 93T449 cells were treated without or with AZD1208 (20 µM) in the absence or presence of the pan-caspase inhibitor z-VAD (50 µM) for 48 h, followed measurement of the number of surviving cells by cell count assay. The cell count assay was done in triplicate. Data are means ± SE of three independent experiments. * *p* < 0.05 compared to the control at the indicated time.

Next, we determined whether treatment with AZD1208 at 20 µM induced apoptosis of 93T449 cells. AZD1208 treatment at 20 µM did not cause nuclear DNA fragmentation at 4, 8 or 24 h (Figure 2A) or an increased accumulation of sub G1 phase cells at 24 h (Figure 2B). Similarly, AZD1208 at 20 µM had no effect on procaspase-9, pro-caspase-3 or PARP expression or cleavage (Figure 2C), while, treatment with z-VAD-fmk, a pan-caspase inhibitor [28], did not interfere with the ability of AZD1208 to reduce survival of 93T449 cells (Figure 2D).

### 2.3. AZD1208 Reduces Phosphorylation of STAT-3 in 93T449 Human Liposarcoma Cells and Pharmacological Inhibition of STAT-3 Leads to Reduction of the Cell Survival

**Figure 3:**
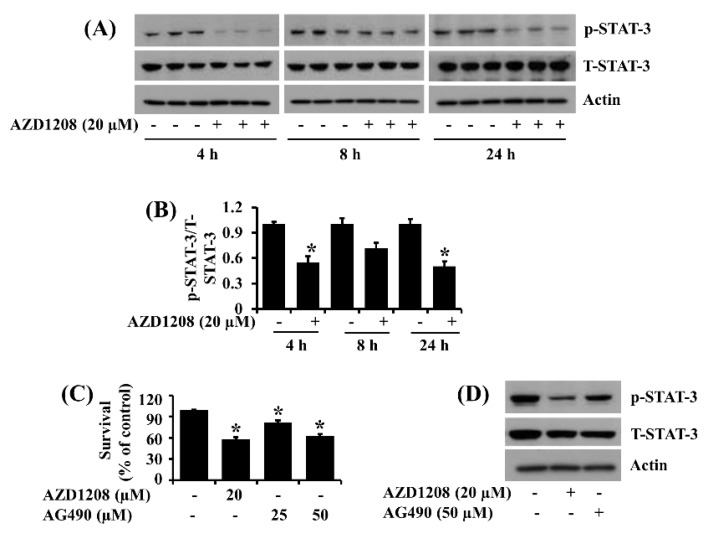
Effect of AZD1208 on expression and phosphorylation levels of STAT-3 in 93T449 cells. (**A**) 93T449 cells were treated with AZD1208 (20 µM) or vehicle control (DMSO) in triplicate experiments for the times designated. At each time point, whole cell lysates were prepared and analyzed for levels of p-STAT-3, T-STAT-3 or β-actin by Western blotting. p-STAT-3, phosphorylated STAT-3; T-STAT-3, total STAT-3. (**B**) The densitometry data of (**A**). * *p* < 0.05 compared to the control at the indicated time. (**C**) 93T449 cells were treated with AZD1208 or AG490, a Jak/STAT-3 inhibitor, at the indicated concentrations for 48 h, followed measurement of the number of surviving cells by cell count assay. The cell count assay was performed in triplicate. Data are means ± SE of three independent experiments. (**D**) 93T449 cells were treated with AZD1208 (20 µM) or AG490 (50 µM) for 24 h. Whole cell lysates from the conditioned cells were prepared and analyzed for p-STAT-3, T-STAT-3 or β-actin by Western blotting. The image is a representative of three independent experiments.

Evidence suggests a role of STAT-3 protein phosphorylation/activation in cancer cell survival [29]. We thus sought to explore whether STAT-3 is expressed and phosphorylated in 93T449 cells and whether AZD1208 modulates STAT-3 protein expression and phosphorylation in the cells. Notably, in the absence of AZD1208 there were substantial expression and phosphorylation of STAT-3 in 93T449 cells at the times tested (Figure 3A). However, treatment with AZD1208 greatly reduced phosphorylation of STAT-3 without affecting its total protein expression in 93T449 cells. The densitometry data of Figure 3A are shown in Figure 3B. Using AG490, a JAK-2/STAT-3 inhibitor, we further determined the role of reduced STAT-3 phosphorylation (activation) in AZD1208’s growth inhibition of 93T449 cells. Similar to AZD1208, AG490 at 25 or 50 µM significantly decreased 93T449 cell survival (Figure 3C) and STAT-3 phosphorylation (Figure 3D). Although there seemed to be a slight decrease in T-STAT-3 expression levels, expression levels of β-actin used as a control for the total proteins loaded remain constant under these experimental conditions.

### 2.4. AZD1208 Inhibits mTOR, S6 and eIF-2α in 93T449 Liposarcoma Cells

**Figure 4:**
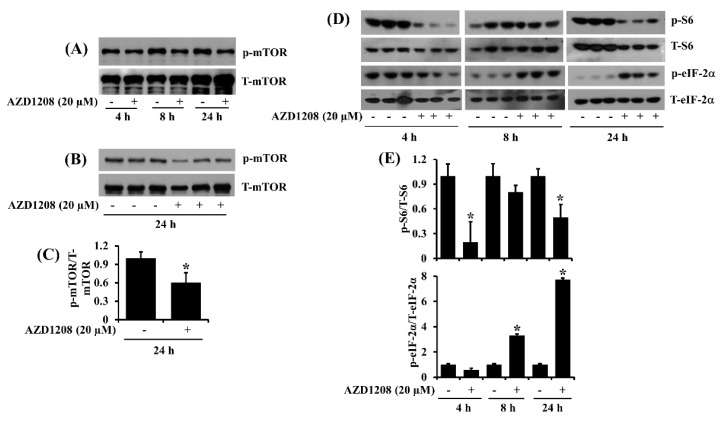
Effect of AZD1208 on expression and phosphorylation of mTOR, S6 and eIF-2α in 93T449 cells. (**A**) 93T449 cells were treated with AZD1208 (20 µM) or vehicle control (DMSO) for the times designated. At each time point, whole cell lysates were prepared and analyzed for p-mTOR and T-mTOR by Western blotting. p-mTOR, phosphorylated mTOR; T-mTOR, total mTOR. (**B**) Western blotting analysis in triplicate experiments for 24 h. (**C**) The densitometry data of (**B**). * *p* < 0.05 compared to the control at the indicated time. (**D**) 93T449 cells were treated with AZD1208 (20 µM) or vehicle control (DMSO) in triplicate experiments for indicated times. At each time point, whole cell lysates were prepared and analyzed for p-S6, T-S6, p-eIF-2α and T-eIF-2α by Western blotting. p-S6, phosphorylated S6; T-S6, total S6; p-eIF-2α, phosphorylated eIF-2α; T-eIF-2α, total eIF-2α. (**E**) The densitometry data of (**D**). * *p* < 0.05 compared to the control at the indicated time.

AZD1208 inhibits growth of acute myeloid leukemia (AML) cells, in part through inhibition of mTOR, S6K, S6 and 4E-BP1 [9]. This prompted us to investigate whether AZD1208 affects the expression and phosphorylation of mTOR, S6 and eIF-2α, translation regulatory proteins, in 93T449 cells. In the absence of AZD1208, there was a time-dependent increase of mTOR phosphorylation in 93T449 cells at the times tested (Figure 4A). Treatment with AZD1208 at 20 µM substantially repressed mTOR phosphorylation without altering its total expression (Figure 4B). The densitometry data of Figure 4B are shown in Figure 4C. Interestingly, there was high phosphorylation of S6 in 93T449 cells at the times tested (Figure 4D). However, treatment with AZD1208 especially at 4 or 24 h resulted in a strong inhibition of S6 phosphorylation in 93T449 cells. Of further note, in the absence of AZD1208, there was high phosphorylation of eIF-2α at 4 h but the protein phosphorylation level largely declined at 8 or 24 h in 93T449 cells. However, treatment with AZD1208 especially at 8 or 24 h led to a significant increase of eIF-2α phosphorylation in 93T449 cells. Total protein expression of S6 and eIF-2α were not largely affected under these experimental conditions. The densitometry data of Figure 4D for S6 and eIF-2α phosphorylation and expression are shown in Figure 4E.

### 2.5. AZD1208 Induces Strong Phosphorylation of AMPK in 93T449 Cells and AMPK Knockdown Greatly Attenuates Reduction of the Cell Survival by AZD1208

**Figure 5:**
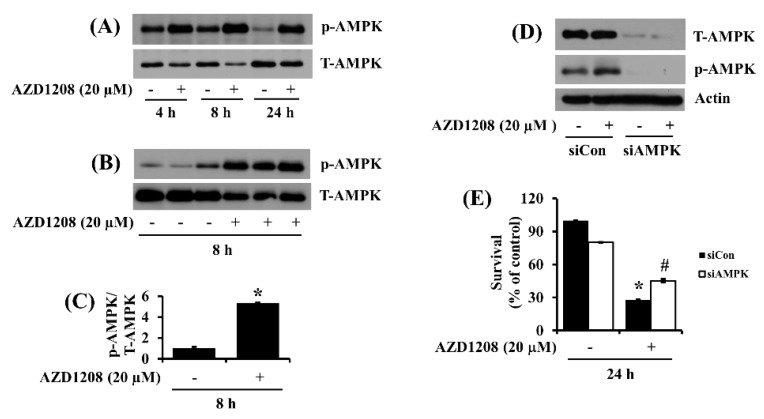
Effect of AZD1208 and/or knock-down of AMPK on survival of 93T449 cells and cellular expression and phosphorylation of AMPK. (**A**) 93T449 cells were treated with AZD1208 (20 µM) or vehicle control (DMSO) for the times designated. At each time point, whole cell lysates were prepared and analyzed for p-AMPK and T-AMPK by Western blotting. p-AMPK, phosphorylated AMPK; T-AMPK, total AMPK. (**B**) Western blotting in triplicate experiments for 8 h. (**C**) The densitometry data of (**B**). * *p* < 0.05 compared to the control at the indicated time. (**D**) 93T449 cells were transfected with 100 pM of control siRNA (siCon) or AMPK siRNA (siAMPK) for 24 h. The siCon- or siAMPK-transfected cells were treated with AZD1208 (20 µM) or vehicle control for 24 h. Whole cell lysates were prepared and analyzed for p-AMPK, T-AMPK or β-actin by Western blotting. (**E**) 93T449 cells were transfected with 100 pM of control siRNA (siCon) or AMPK siRNA (siAMPK) for 24 h. The siCon- or siAMPK-transfected cells were treated with AZD1208 (20 µM) or vehicle control for 24 h, followed measurement of the number of surviving cells by cell count assay. The cell count assay was performed in triplicate. Data are means ± SE of three independent experiments. * *p* < 0.05 compared to the control at the indicated time; # *p* < 0.05 compared to AZD1208 at the indicated time.

Recently, we demonstrated that AZD1208 induces a strong phosphorylation of AMPK during the differentiation of 3T3-L1 preadipocytes into adipocytes [25]. We thus investigated whether AMPK is expressed and phosphorylated in 93T449 cells and whether AZD1208 modulates it. Time course experiments revealed that there was substantial phosphorylation of AMPK in 93T449 cells at 4 or 8 h but the protein phosphorylation level largely declined at 24 h. However, at all times tested, treatment with AZD1208 resulted in strong further phosphorylation of AMPK (Figure 5A). Triplicate experiment further confirmed that 8 h treatment with AZD1208 significantly up-regulated AMPK phosphorylation in 93T449 cells (Figure 5B). The densitometry data of Figure 5B are shown in Figure 5C. Using control or AMPK siRNA transfection, we next determined the role of AMPK activation (phosphorylation) in AZD1208’s growth inhibitory effect in 93T449 cells. As shown in Figure 5D, there were much lower protein expression and phosphorylation of AMPK in AMPK siRNA (siAMPK)-transfected 93T449 cells compared with control siRNA (siCon)-transfected ones, showing efficient siAMPK transfection and knockdown. As shown in Figure 5E, in the absence of AZD1208, there was a slight reduction of survival of the siAMPK-transfected 93T449 cells compared with siCon-transfected controls. However, in the presence of AZD1208, there was a much higher survival of the siAMPK-transfected 93T449 cells compared with siCon-transfected controls.

### 2.6. AZD1208-Induced AMPK Phosphorylation in 93T449 Cells Appears to Be Not through LKB-1 But through Decrease in Cellular ATP Contents

**Figure 6:**
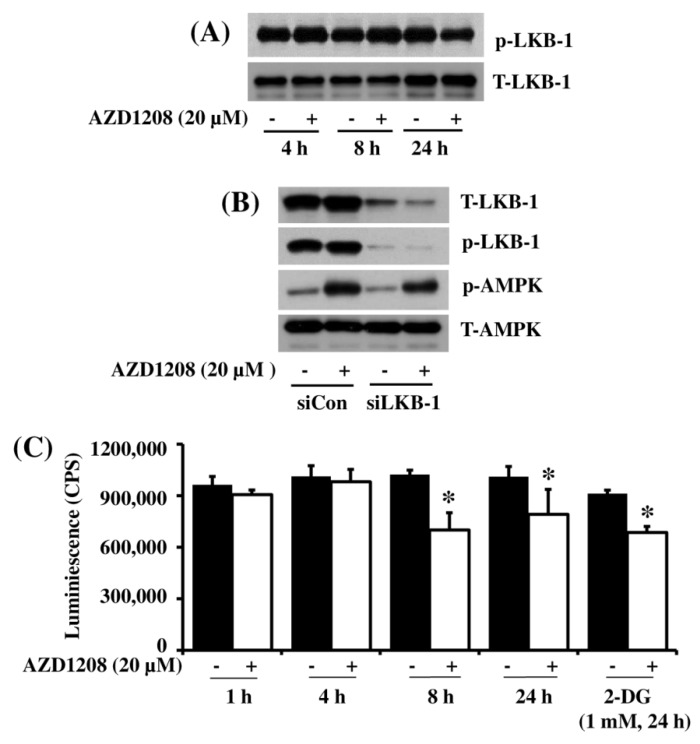
Effect of AZD1208 and/or knock-down of LKB-1 on expression and phosphorylation of LKB-1 and AMPK and cellular ATP content in 93T449 cells. (**A**) 93T449 cells were treated with AZD1208 (20 µM) or vehicle control (DMSO) for the times designated. At each time point, whole cell lysates were prepared and analyzed for p-LKB-1 and T-LKB-1 by Western blotting. p-LKB-1, phosphorylated LKB-1; T-LKB-1, total LKB-1. The image is a representative of three independent experiments. (**B**) 93T449 cells were transfected with 100 pM of control siRNA (siCon) or LKB-1 siRNA (siLKB-1) for 24 h. The siCon- or siAMPK-transfected cells were treated with AZD1208 (20 µM) or vehicle control for 24 h. Whole cell lysates were prepared and analyzed for T-LKB-1, p-LKB-1, p-AMPK and T-AMPK by Western blotting. (**C**) 93T449 cells were treated with AZD1208 (20 µM) or vehicle control for the times designated. For comparison, cells were treated with 1 mM of deoxyglucose (2-DG), a known ATP depleting agent for 24 h. At each time point, cellular ATP content was measured by ATP measurement kit. * *p* < 0.05 compared to the value of AZD1208 or 2-DG free control at the indicated time.

Increasing evidence suggests that AMPK phosphorylation is greatly regulated by LKB-1 [30]. We thus investigated whether LKB-1 is expressed and phosphorylated in 93T449 cells and whether AZD1208 modulates it. In the absence of AZD1208, there were high phosphorylation and expression of LKB-1 in 93T449 cells at all the times tested (Figure 6A). Interestingly, 4 or 8 h treatment with AZD1208 further enhanced LKB-1 phosphorylation without affecting its total expression in 93T449 cells. However, AZD1208 treatment at 24 h largely decreased LKB-1 phosphorylation without affecting its total expression in 93T449 cells. Using control siRNA (siCon) or LKB1 siRNA (siLKB-1) transfection, we next investigated the role of LKB-1 in AZD1208-induced AMPK phosphorylation in 93T449 cells. There were much lower expression and phosphorylation of LKB-1 in siLKB-1-transfected 93T449 cells compared with siCon-transfected ones, showing the siLKB-1 transfection and knockdown efficiency (Figure 6B). However, in the presence of siLKB-1 knockdown, there still remained a strong AMPK phosphorylation. Moreover, there was no big difference in AMPK phosphorylation between siCon- and siAMPK-transfected cells. Given that AMPK activation is also controlled by change in the cellular AMP/ATP ratio [31], we next determined whether AZD1208 alters the amount of cellular ATP in 93T449 cells. For comparison, 2-deoxyglucose (2-DG) that lowers cellular ATP content [32] was included as a positive control. As expected, 24 h treatment with 2-DG at 1 mM substantially lowered cellular ATP content in 93T449 cells (Figure 6C). Similarly, 8 or 24 h treatment with AZD1208 also decreased cellular ATP content in the cells.

### 2.7. AZD1208 Does Not Affect Expression of Pim Kinases but It Reduces the Phosphorylation of 4EBP-1, a Downstream Effector of Pim Kinases, in 93T449 Human Liposarcoma Cells

**Figure 7:**
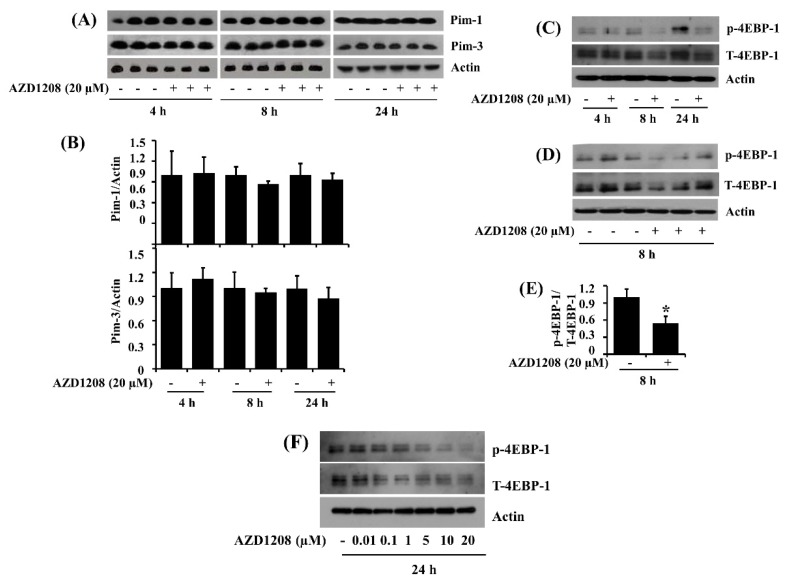
Effect of AZD1208 on expression and/or phosphorylation of Pim kinases and 4EBP-1 in 93T449 cells. (**A**) 93T449 cells were treated with AZD1208 (20 µM) or vehicle control (DMSO) in triplicate experiments for the times designated. At each time point, whole cell lysates were prepared and analyzed for Pim-1, Pim-3 and β-actin by Western blotting. (**B**) The densitometry data of (**A**). (**C**) 93T449 cells were treated with AZD1208 (20 µM) or vehicle control for the times designated. At each time point, whole cell lysates from the conditioned cells were prepared and analyzed for p-4EBP-1, T-4EBP-1 and β-actin by Western blotting. p-4EBP-1, phosphorylated 4EBP-1; T-4EBP-1, total 4EBP-1. (**D**) Western blotting in triplicate experiments for 8 h. (**E**) The densitometry data of (**D**). * *p* < 0.05 compared to the value of AZD1208 free control at the indicated time (**F**) 93T449 cells were treated with different concentrations of AZD1208 (0.01, 0.1, 1, 5, 10 and 20 µM) or vehicle control for 24 h. Whole cell lysates from the conditioned cells were prepared and analyzed for p-4EBP-1, T-4EBP-1 and β-actin by Western blotting. p-4EBP-1, phosphorylated 4EBP-1; T-4EBP-1, total 4EBP-1.

Given that AZD1208 is a pan-Pim kinase inhibitor, we next examined whether members of Pim kinase family are expressed in 93T449 cells and whether AZD1208 (20 μM) modulates their expression and/or kinase activity. Notably, as shown in Figure 7A, in the absence of AZD1208, there was substantial expression of Pim-1 and Pim-3 but not Pim-2, in 93T449 cells. The densitometry data of Figure 7A for Pim-1 and Pim-3 protein expression are shown in Figure 7B. There was also an enhancement of phosphorylation of 4EBP-1, a known downstream substrate of Pim kinases [33], in 93T449 cells at all the times tested (Figure 7C). However, treatment with AZD1208 at 20 μM repressed 4EBP-1 phosphorylation in 93T449 cells (Figure 7C,D). The densitometry data of Figure 7D are shown in Figure 7E. Given that AZD1208 is a low-nanomolar inhibitor of Pim kinase, we further determined the potential concentration of AZD1208 required to inhibit Pim kinases in 93T449 cells. Of note, treatment with AZD1208 at lower concentrations applied (0.01, 0.1 or 1 μM) does not inhibit phosphorylation of 4EBP-1, whereas the drug treatment at 5, 10 or 20 μM markedly inhibits 4EBP-1 phosphorylation in 93T449 cells (Figure 7F). Apparently, 20 μM of AZD1208 appeared to maximally inhibit 4EBP-1 phosphorylation in 93T449 cells. These results strongly suggest that the growth inhibition of 93T449 liposarcoma cells by AZD1208 at 20 μM is attributable to Pim kinases inhibition.

### 2.8. Pim-3 Knock-Down Causes Not Only Growth Inhibition But Also Alteration of Phosphorylation of 4EBP-1, STAT-3, mTOR, AMPK and eIF-2a in 93T449 Human Liposarcoma Cells

**Figure 8:**
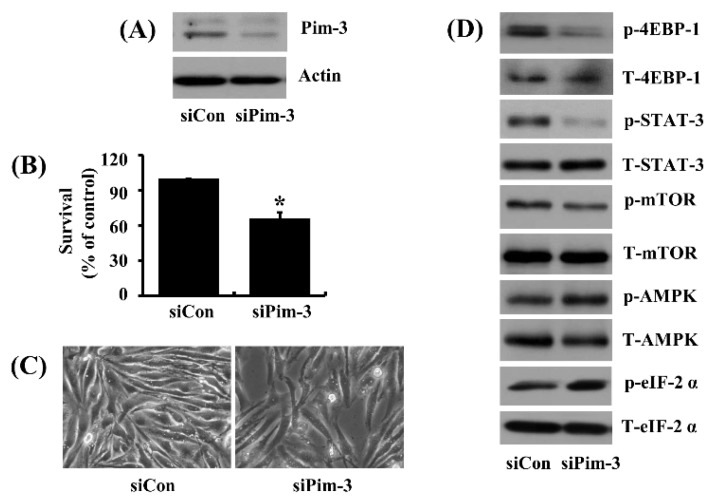
Effect of Pim-3 knock-down on survival of 93T449 cells and cellular expression and/or phosphorylation of 4EBP-1, STAT-3, AMPK, mTOR and eIF-2α. (**A**) 93T449 cells were transfected with 100 pM of control siRNA (siCon) or Pim-3 siRNA (siPim-3) for 24 h. Whole cell lysates were prepared and analyzed for Pim-3 and β-actin by Western blotting. (**B**) 93T449 cells were transfected with 100 pM of control siRNA (siCon) or Pim-3 siRNA (siPim-3) for 24 h, followed measurement of the number of surviving cells by cell count assay. The cell count assay was performed in triplicate. Data are means ± SE of three independent experiments. * *p* < 0.05 compared to the siConc at the indicated time. (**C**) Images of the siCon- or siPim-3-transfected cells were obtained by phase contrast microscopy, 400 ×. (**D**) 93T449 cells were transfected with 100 pM of control siRNA (siCon) or Pim-3 siRNA (siPim-3) for 24 h. Whole cell lysates were prepared and analyzed for p-4EBP-1, T-4EBP-1, p-STAT-3, T-STAT-3, p-AMPK, T-AMPK, p-mTOR, T-mTOR, p-eIF-2α and T-eIF-2α by Western blotting.

We further probed the role of Pim-3 in AZD1208-induced AMPK phosphorylation in 93T449 cells using control siRNA (siCon) or Pim-3 siRNA (siPim-3) knockdown. siPim-3-transfection led to an efficient knockdown of Pim-3 in 93T449 cells compared with siCon-transfected ones (Figure 8A). Notably, cell count analysis and microscopic observation demonstrated decreased cell survival in siPim-3-knockdown 93T449 cells compared with siCon-transfected ones (Figure 8B,C), similar to what we observed with AZD1208 treatments. Additionally and again identical to AZD1208, siPim-3-knockdown led to a lower phosphorylation of 4EBP-1, STAT-3 and mTOR and an increased phosphorylation of AMPK and eIF-2α when compared with siCon-transfected cells (Figure 8D).

## 3. Discussion

Pim kinases play important roles in proliferation, survival and growth of a number of hematological and solid cancers, supporting Pim kinases as valid therapeutic targets [8,10,33,34,35]. Indeed, numerous Pim kinases inhibitors have shown their anti-cancer property in multiple tumor models in vitro and in vivo [9,36,37]. AZD1208 is a pan-Pim kinase inhibitor that is known to have potent anti-cancer activity in blood cancer [9,10] and prostate cancer cells [21]. AZD1208 has yet to be investigated in adipocytic tumors. In this study, we report for the first time that AZD1208 inhibits the growth of 93T449 human liposarcoma cells through Pim-3, 4EBP-1, mTOR, S6, eIF-2α, STAT-3 and AMPK. We further provide evidence that Pim-3 is crucial for the growth of 93T449 cells and the phosphorylation (activity) of 4EBP-1, STAT-3, AMPK, mTOR and eIF-2α.

AZD1208 inhibits proliferation and growth of AML cell lines [9,10] and induces apoptosis in prostate cancer graft specimens [21]. In the 93T449 human liposarcoma, AZD1208 at 20 µM was highly cytostatic (cytotoxic) but not apoptotic, as evidenced by cell death with no increase in any apoptotic marker measured: sub G1 phase, nuclear DNA fragmentation or caspase-9/3 activation. These results support the notion that AZD1208-mediated growth inhibition in 93T449 cells at 20 μM is not through classical apoptosis but through regulation of other mechanisms and/or factors.

The family of STATs plays a crucial role in the expression of genes which are involved in cancer cell survival, proliferation, apoptosis and angiogenesis [29,38,39]. Among the STAT family, STAT-3 has been extensively studied due to its constitutive expression in many human cancers including soft tissue sarcoma cells [40,41,42]. Importantly, mounting evidence indicates that STAT-3 activation contributes to proliferation and oncogenesis by modulating the expression of a variety of genes required for tumor cell survival, proliferation and angiogenesis, as well as invasion and metastasis [43] but STAT-3 inhibition leads to suppression of the growth of numerous cancers in vitro and in vivo [29,44,45]. It is thus suggested that targeted disruption of STAT-3 could be one potential approach to treat human cancers overexpressing STAT-3 [46]. Until now, little is known about the phosphorylation of STAT-3 and AZD1208 regulation of the STAT-3 phosphorylation in human liposarcoma cells. In this study, STAT-3 was highly phosphorylated in 93T449 cells and AZD1208 at 20 μM greatly inhibited this STAT-3 phosphorylation. In addition, pharmacological inhibition of STAT-3 by AG490, a JAK2/STAT-3 inhibitor, leads to partial growth inhibition of 93T449 cells. These results suggest that STAT-3 activity is necessary for the survival of 93T449 cells and that STAT-3 inhibition contributes to the growth inhibition effects of AZD1208 at 20 μM in 93T449 cells.

AZD1208 inhibits phosphorylation of 4EBP1, p70S6K and S6 and suppresses translation in MOLM-16 AML cells [18]. Of further note, there is a recent study demonstrating that AZD1208 suppresses mTOR signaling, including inhibition of phosphorylation of mTOR (S2448), p70S6K (T389), S6 (S235/236) and 4EBP-1 (S65), in AML cell lines, which contribute to AZD1208’s cytostatic effects [9]. Moreover, there is further evidence that Pim kinases regulate mTOR activity [47]. mTOR, S6K, S6 and 4EBP-1 are key proteins involved in translation [9]. Accordingly, mTOR regulates protein synthesis by hyperphosphorylating 4EBP-1, which decreases its affinity for the translation initiation factor eIF4E [48] and phosphorylating and activating p70S6K, which in turn phosphorylates the 40S ribosomal subunit S6 protein [49]. We showed in this study that AZD1208 at 20 μM does not alter expression of Pim-1/3 but it greatly inhibits phosphorylation of mTOR, S6 and 4EBP-1 in 93T449 cells, suggesting that the AZD1208’s cytostatic effect in 93T449 cells at 20 μM is attributable to by inhibition of mTOR, S6 and 4EBP-1 proteins, thereby leading to suppression of translation in 93T449 cells. A notable finding of the present study is AZD1208 regulation of eIF-2α protein. Accordingly, eIF-2α is phosphorylated in response to cellular stress and its phosphorylation leads to global translation inhibition by suppressing the formation of ternary complex (eIF2-GTP-Met-tRNA) in translation initiation [50]. Thus considering that AZD1208 largely up-regulates eIF-2α phosphorylation in 93T449 cells, it is conceivable that the AZD1208-induced eIF-2α phosphorylation will further interfere with translation initiation in 93T449 cells, which would facilitate the drug’s cytostatic effect.

AMPK is a key regulator of energy metabolism and balance [51]. It is a heterotrimeric protein kinase consisting of a catalytic α subunit and regulatory β and γ subunits. Mounting evidence strongly suggests that AMPK activation leads to cell cycle arrest and growth inhibition in several tumor cells [10,52,53,54,55]. Interestingly, the Pim-mediated regulation of AMPK activity has been previously proposed, with the facts that a Pim kinase inhibitor SMI-4a induces activation of AMPK via increased AMP/ATP ratio in mouse embryonic fibroblasts [10]. Recently, we also have demonstrated the ability of AZD1208 to induce strong AMPK phosphorylation in 3T3-L1 preadipocytes [25]. At present, little is known about AZD1208 regulation of AMPK in liposarcoma cells. Strikingly, we observed that AZD1208 at 20 μM greatly increases AMPK phosphorylation in 93T449 cells and knock-down of AMPK largely attenuates the ability of AZD1208 to inhibit growth of 93T449 cells. These results strongly indicate that the AZD1208’s cytostatic effect on 93T449 cells is largely due to AMPK activation. It is documented that AMPK phosphorylation is controlled by upstream kinases, such as LKB-1 [30] and change in cellular ATP content [31]. Our findings showed that AMPK activation by AZD1208 in 93T449 cells was LKB-1-independent but linked rather to a reduced cellular ATP content. Cellular expression of Pim kinases is regulated at multiple stages, including transcription, post-transcription and translation [8]. Because Pim kinases are constitutively expressed and active with very short protein half-life (<5 min), it is suggested that regulation of the protein stability is critical for their cellular function and activity [56]. It is of interest that only Pim-1 and Pim-3 but not Pim-2, were highly expressed in 93T449 cells. These results suggest 93T449 cells contain unique regulatory systems/factors that selectively lead to high protein expression and/or stability of Pim-1 and Pim-3. AZD1208 at 20 µM did not affect expression of Pim-1 and Pim-3 but strongly reduced phosphorylation of 4EBP-1, a well-characterized downstream substrate of Pim kinases in 93T449 cells. Further considering that, the 1 μM concentration of AZD1208 inhibiting cell growth does not inhibit phosphorylation of 4EBP-1 in 93T449 cells. The cytostatic response of 93T449 cells to AZD1208 at 20 μM appears to be in part attributable to Pim kinases inhibition and also mediated via inhibition of several off-targets, such as STAT-3, AMPK, mTOR and eIF-2α herein. As of now, little is known about the role of Pim kinases in liposarcoma. Here we demonstrated that Pim-3 expression and activity are crucial for not only the growth of 93T449 cells but also the phosphorylation (activity) of mTOR, 4EBP-1, eIF-2α, STAT-3 and AMPK, given that knockdown of Pim-3 leads to growth inhibition of 93T449 cells, decrease in phosphorylation of 4EBP-1, STAT-3 and mTOR but increase in phosphorylation of AMPK and eIF-2α in the cells. It further suggests that Pim-3 lies upstream of mTOR, 4EBP-1, eIF-2α, STAT-3 and AMPK in 93T449 cells.

In summary, this is the first study reporting that AZD1208 has a cytostatic effect in human liposarcoma cells, which is be mediated through control of the expression and/or phosphorylation of Pim kinases, 4EBP-1, mTOR, S6, eIF-2α, STAT-3 and AMPK. Although there are still important issues that remain to be resolved, including anti-tumor effect of AZD1208 on animal models, the present findings support that AZD1208 is a potential therapeutics for the treatment of liposarcoma.

## 4. Materials and Methods

### 4.1. Chemicals and Antibodies

AZD1208 was purchased from Selleckchem (Houston, TX, USA). RPMI-1640 (LM011-01), DMEM (LM001-05), fetal bovine serum (FBS) (S001-01) and cocktail of penicillin/streptomycin antibiotics (LS202-02) were obtained from WelGENE (Daegu, Korea). Anti-procaspase-9 (ADI-AAM-139) and anti-procaspase-3 (ADI-AAP-113) antibodies were bought from Enzo (Farmingdale, NY, USA). Anti-PARP (11835238001) antibody was obtained from Roche Diagnostics (Mannheim, Germany). Anti-actin (A5441) antibody was purchased from Sigma (St. Louis, MO, USA). Anti-phospho(p)-4EBP-1 (S65) (#9455), anti-4EBP-1 (#9452), anti-p-S6 (S235/236) (#2211), anti-S6 (#2317), anti-p-mTOR (S2448) (#5536), anti-mTOR (#2972), anti-p-AMPK (T172) (#2535), anti-AMPK (#2793), anti-p-LKB-1 (S428) (#3482), anti-LKB-1(#3047) and anti-eIF-2α (#9722), antibodies were purchased from Cell Signaling Technology (Danvers, MA, USA). Anti-p-eIF-2α (S51) (ab32157) antibody was bought from Abcam (Cambridge, MA, USA). Anti-Pim-1 (sc-374116), anti-Pim-3 (sc-98959), anti-p-STAT-3 (Tyr(Y)705) (sc-8059), anti-STAT-3 (sc-8019), secondary goat anti-Rabbit and goat anti-Mouse IgG antibodies, control siRNA (sc-37007), AMPK siRNA (sc-45312), LKB-1 siRNA (sc-35816) and Pim-3 siRNA (sc-61353) were purchased from Santa Cruz Biotechnology (Delaware, CA, USA). z-VAD-fmk and AG490 were purchased from Calbiochem (Madison, WI, USA). Super Signal™ West Pico PLUS Enhanced chemiluminescence (ECL, #34080) was purchased from Thermo Scientific (Waltham, MA, USA). Plasticwares: 6-well and 24-well plates and 60 and 100 mm of cell culture dish were obtained from SPL Life Sciences (Gyeonggi-do, Korea).

### 4.2. Cell Culture

Human 93T449 (CRL-3043™) and SW872 (CRL-HTB92™) liposarcoma cells (ATCC; Manassas, VA, USA) were grown in RPMI 1640/DMEM media supplemented with 10% heat-inactivated FBS (HI-FBS) and 1% penicillin/streptomycin at 37 °C in a humidified condition of 95% air and 5% CO_2_.

### 4.3. Cell Count Assay and Cell Morphology Analysis

93T449 and SW872 cells were seeded in a 24-well plate (0.5 × 10^5^ cells/500 µL/well) overnight. Cells were treated without or with AZD1208 or other agents (AG490, z-VAD-fmk) at the indicated concentrations and for the times designated. At each time point, the number of surviving cells, which cannot be stained with trypan blue dye, was counted using phase contrast microscope. Approximately 50 cells were counted for each evaluation. The cell count assay was performed in triplicate. Data are means ± standard errors (SE) of three independent experiments. Survival is expressed as a percentage of control. For cell morphology analysis, phase contrast images of the conditioned cells treated with AZD1208 or transfected with siRNA (control, Pim-3) were taken with a compound microscope (Nikon Eclipse TS200, Nikon Corp., Tokyo, Japan).

### 4.4. Measurement of DNA Fragmentation

Measurement of DNA fragmentation was conducted as mentioned in our previous study [57]. Briefly, 93T449 cells were seeded at a density of (2 × 10^5^ cells/mL) the day before treatment. Cells were treated with AZD1208 (20 µM) or vehicle control (DMSO) for 4, 8 or 24 h. At each time point, the conditioned cells were harvested, washed and lysed in a buffer [50 mM Tris (pH 8.0), 0.5% sarkosyl, 0.5 mg/mL proteinase K and 1 mM EDTA] at 55 °C for 3 h, followed by the addition of RNase A (0.5 μg/mL) and incubation at 55 °C for 18 h. The lysate was centrifuged at 10,000× *g* for 20 min. Genomic DNA was extracted with equal volume of neutral phenol-chloroform-isoamyl alcohol mixture (25:24:1) and analyzed by electrophoresis on a 1.8% agarose gel. The DNA was visualized and photographed under UV illumination after staining with ethidium bromide (0.1 µg/mL) by Gel documentation system (Gel Doc-XR, Bio-rad, Hercules, CA, USA).

### 4.5. Measurement of the Population of Sub G1 Phase by Flow Cytometry Analysis

After 24 h treatment with vehicle control (DMSO) or AZD1208 (20 µM), 93T449 cells were harvested and washed with PBS, fixed in ice-cold 70% ethanol and stored at 4 °C. Prior to analysis, cells were again washed once with PBS, suspended in 1 mL of cold propidium iodide solution containing 100 μg/mL RNase A, 50 μg/mL propidium iodide, 0.1% (*w*/*v*) sodium citrate and 0.1% (*v*/*v*) NP-40 and further incubated on ice for 30 min in the darkness. Cytometric analyses were carried out with a flow cytometer (FACS Caliber, Becton Dikinson, MD, USA) and CellQuest software. Approximately, 10,000 cells were counted for the analysis.

### 4.6. Preparation of Whole Cell Lysates

93T449 cells were seeded in 6-well plates the day before treatment. Cells were treated with AZD1208 (20 μM) and/or other reagents or vehicle control (DMSO) for the indicated times. At each time point, cells were washed twice with PBS and proteins were extracted using modified RIPA buffer [50 mM Tris-Cl (pH 7.4), 150 mM NaCl, 0.1% sodium dodecyl sulfate, 0.25% sodium deoxycholate, 1% Triton X-100, 1% Nonidet P-40, 1 mM EDTA, 1 mM EGTA, proteinase inhibitor cocktail (1X)]. The cell lysates were collected and centrifuged at 12,000 rpm for 20 min at 4 °C. The supernatant was saved and its protein concentration was determined by bicinchoninic acid assay (BCA) Protein Assay Kit (Thermo scientific, Rockford, IL, USA).

### 4.7. Immunoblot Analysis

An equal amount of proteins (50 μg) was separated by SDS-PAGE and transferred onto polyvinyldenefluoride (PVDF) membrane (Millipore; Billerica, MA, USA) by electroplating. The membranes were washed with Tris-buffered saline (TBS) (10 mM Tris, 150 mM NaCl, pH 7.5) supplemented with 0.05% (*v*/*v*) Tween 20 (TBS-T) followed by blocking with TBS-T containing 5% (*w/v*) non-fat dried milk. The membranes were probed overnight with antibodies specific for the protein of interest at 4 °C, followed by incubating with secondary antibodies coupled to horseradish peroxidase at room temperature for 2 h. The membranes were washed and immune-reactivities were detected by Super Signal™ West Pico PLUS enhanced chemiluminescence (ECL) according to manufacturer (Thermo Scientific, Waltham, MA, USA). Equal protein loading was assessed by the expression levels of β-actin.

### 4.8. Small Interfering RNA (siRNA) Transfection

93T449 cells (1 × 10^5^ cells/mL) were seeded into 6-well plates and transfected for 6 h with 100 picomole (pM) of control, AMPK, LKB-1 or Pim-3 siRNA using Lipofectamine^®^ RNAiMAX Transfection Reagent (Invitrogen, Waltham, MA, USA). Culture media from the conditioned cells were removed and refreshed with RPMI-1640 containing 10% HI-FBS, followed by incubation for 18 h. After 24 h post-transfection, the conditioned cells were treated with or without AZD1208 for the designated times. The numbers of surviving cells, which cannot be stained with trypan blue dye, were counted under microscope. The cell count assay was done in triplicates. Data are means ± SE of three independent experiments. Whole cell lysates were also prepared from the conditioned cells and analyzed for Western blotting.

### 4.9. Measurement of Cellular ATP Contents

93T449 cells (0.3 × 10^5^ cell per well) were seeded in 96 well-plates and treated without or with AZD1208 (20 μM) or 2-deoxyglucose (2-DG), a glucose mimetic that depletes levels of cellular ATP, for the indicated times and doses. At each time point, cellular ATP levels were measured by luciferase activity using a luminescence assay kit according to the manufacturer’s protocol (#6016941, ATPLite-1step, Perkin Elmer Inc., Waltham, MA, USA). After 2 min incubation, luminescence was measured on a Victor (Perkin Elmer).

### 4.10. Statistical Analyses

Cell count analysis was performed in triplicates and repeated three times. Data were expressed as mean ± SE. The significance of difference was determined by One-Way ANOVA (Laerd Statistics, Chicago, IL, USA). All significance testing was based upon a *p* < 0.05.

## Abbreviations

ECL	enhanced chemiluminescence
mTOR	mammalian target of rapamycin
FBS	fetal bovine serum
STAT-3	signal transducer and activator of transcription-3
e-IF-2α	eukaryotic initiation factor-2α
RT	room temperature
